# Olfactory decline profiles and their association with dementia conversion in an older population with 12 years of follow‐up

**DOI:** 10.1002/alz.092262

**Published:** 2025-01-03

**Authors:** Ingrid Ekström, Bárbara Avelar‐Pereira, Robert Ruane, Giulia Grande, Laura Fratiglioni, Maria Larsson, Erika J Laukka

**Affiliations:** ^1^ Aging Research Center, Karolinska Institutet and Stockholm University, Stockholm Sweden; ^2^ Stanford University, Stanford, CA USA; ^3^ Stockholm Gerontology Research Center, Stockholm Sweden; ^4^ Gösta Ekman Laboratory, Stockholm University, Stockholm Sweden

## Abstract

**Background:**

Olfactory impairment represents an early sensory marker of neurodegeneration, but little is known about how interindividual differences in the trajectories of olfactory change relate to dementia conversion. We aimed to characterize olfactory decline profiles during phases of normal cognition to assess their predictive value for dementia conversion.

**Method:**

Olfactory trajectory profiles of dementia‐free persons (SNAC‐K; n = 1601; mean age = 70.0 years) were identified using growth mixture models (GMM) of repeated assessments with the Sniffin’ sticks odor identification task over 6 years of follow‐up. Olfactory functioning for old‐old participants (≥78 years) was assessed three times (at 3‐year‐interval), whereas young‐old participants (<78) were assessed twice (6‐year‐interval). Hazard ratio (HR) of dementia occurrence across 6 years after the olfactory trajectories were established was estimated with Cox regression. In total, participants were followed for up to 12 years.

**Result:**

GMM analysis identified three distinct olfactory trajectory profiles. As opposed to the “maintained olfaction” profile (n = 952), the “decline” (n = 433) and “accelerated decline” (n = 216) profiles were characterized by lower olfactory ability at baseline, a more rapid decline over follow‐up, and were associated with increased hazard of dementia (HR: 1.82; 95% confidence interval [CI] 1.12‐2.96 for “decline” and HR: 3.55; 95% CI: 2.09‐6.02 for ”accelerated decline”). Sensitivity analyses identified two olfactory trajectory profiles for old‐old (i.e., maintain and decline) and four trajectory profiles for young‐old participants (i.e. maintain, decline, accelerated decline, and pronounced decline). Associations between low‐performing olfactory trajectory profiles and dementia were particularly strong in young‐old participants with “pronounced decline” (n = 66, HR: 9.52; 95% CI:2.54‐35.72).

**Conclusion:**

We identified profiles based on olfactory decline trajectories in a sample that was initially cognitively normal. Participants with lower baseline olfaction and more rapid olfactory decline were grouped together, and these profiles were associated with an increased risk of developing dementia, as compared to participants who maintained their olfactory ability. Our results point towards a relatively early onset of dementia‐related olfactory decline. Intact olfactory ability in older age may indicate low risk of dementia conversion. As such longitudinal screening of olfaction may offer prognostic value.

